# Association Between Smoking and Stroke: A Retrospective Study Using the 2022 Behavioral Risk Factor Surveillance System (BRFSS) Data

**DOI:** 10.7759/cureus.89171

**Published:** 2025-07-31

**Authors:** Saow Renn Ding, Khushi Patel, Sai Praneeth Chaparala, Pujitha Pendyala, Khushboo Rani

**Affiliations:** 1 Medicine, St. George’s University School of Medicine, St. George, GRD; 2 Internal Medicine, Surat Municipal Institute of Medical Education and Research (SMIMER), Surat, IND; 3 Internal Medicine, Gayatri Vidya Parishad Institute of Health Care and Medical Technology, Visakhapatnam, IND; 4 Medicine, Sri Venkata Sai (SVS) Medical College, Mahabubnagar, IND; 5 Internal Medicine, Goa Medical College and Hospital, Bambolim, IND

**Keywords:** brfss, demographic factor, smoking, socioeconomic status, stroke

## Abstract

Introduction: Stroke is the leading cause of disability and death worldwide, often resulting from disrupted blood flow to the brain. Smoking is a well-established risk factor that significantly increases the likelihood of stroke by damaging blood vessels and promoting clot formation.

Aim and objective: This study aimed to assess the association between smoking status and self-reported stroke, considering demographic and socioeconomic factors.

Methodology: This retrospective study was conducted using the 2022 Behavioral Risk Factor Surveillance System (BRFSS) data to assess the relationship between smoking and self-reported stroke. Demographic (age, gender, and race) and socioeconomic (education and income) factors were included as controls. Data were analyzed using cross-tabulations, with results reported as odds ratios (ORs) and 95% confidence intervals (CIs).

Results: This study indicates that smokers are 1.71 times more likely to experience a stroke compared to non-smokers (95% CI: 1.6498-1.7839). Among individuals aged 18-24, smokers have a 5.2-fold higher risk of stroke than non-smokers in the same age group. Female smokers are 2.8 times more likely to suffer a stroke compared to their non-smoking counterparts. When analyzed by race, Hispanic smokers show a higher risk of stroke than Hispanic non-smokers. Additionally, smokers with advanced education levels are more prone to strokes than equally educated non-smokers. Those earning less than $50,000 annually also face a greater stroke risk if they smoke, compared to non-smokers within the same income bracket.

Conclusions: Smoking is significantly associated with increased risk of stroke, especially among young adults, women, Hispanic race, the highly educated, and those with lower income. These results underscore the need for targeted stroke prevention efforts among high-risk smoking populations.

## Introduction

Stroke is a medical condition characterized by the sudden onset of localized neurological impairment resulting from vascular damage, either due to infarction or hemorrhage, affecting the central nervous system [1]. After ischemic heart disease, stroke was the second leading cause of mortality worldwide in 2016 (5.5 million fatalities; 95% CI: 5.3-5.7). Compared to men (2.9 million fatalities; 2.8-3.0), women experienced fewer stroke-related deaths (2.6 million; 2.5-2.7). Globally, ischemic stroke caused slightly fewer deaths (2.7 million; 2.6-2.8) than hemorrhagic stroke (2.8 million; 2.7-2.9) [2]. Stroke risk factors include both modifiable ones, like diet and medical conditions, and non-modifiable ones, such as age and race. These can also be classified by timeframe: short-term triggers (like infections or stress), intermediate risks (like high blood pressure or cholesterol), and long-term factors (such as sex and race) [3].

Smoking is widespread globally, with particularly high rates among young people and in developing countries [4]. Multiple studies have been carried out recently to investigate the effects of smoking on stroke [5,6]. Structural artery wall damage and carotid atherosclerosis, which result in thrombosis or embolic events, are two mechanisms that connect smoking to ischemic stroke [7,8]. The short-term effects of smoking, known as thrombogenesis, include raised hematocrit levels, decreased cerebral blood flow due to arterial vasoconstriction, and increased fibrinogen and platelet aggregability [9-11]. The observed decrease in risk of ischemic stroke following quitting smoking supports the significance of these variables in causing stroke in smokers [12]. Based on recent evidence, smoking is closely linked to inflammatory factors, which are crucial in the pathophysiology of stroke [13].

The Behavioral Risk Factor Surveillance System (BRFSS) is a state-based nationwide telephone survey program in the US that gathers information on chronic illnesses, health-related risk behaviors, and the utilization of preventative interventions. [14].

Aims and objectives

This study aimed to evaluate the association between smoking and stroke by assessing the odds of developing stroke in smokers versus non-smokers, based on the demographic (age, gender, race) and socioeconomic characteristics (annual income and education levels) of participants.

## Materials and methods

A retrospective original research study was conducted using the BRFSS database [15]. Data were extracted on May 18, 2025. This study involved non-human participants, as the BRFSS database contains de-identified publicly available data. Therefore, no ethics committee approval was required.

Data was extracted from the BRFSS Web Enabled Analysis Tool (WEAT), focusing on the year 2022. The primary variable of interest in this study was the disease variable stroke, “Ever told you had stroke (CVDSTRK3)”, and the lifestyle risk factor variable smoking, “Calculated variable for adults who are current smokers (_RFSMOK3)”. The study included several control variables to adjust for potential confounders, ensuring a comprehensive analysis. Demographic characteristics were carefully considered, including age (_AGE_G: 18-24, 25-44, 45-64, 65+), gender (respondents' sex, categorized as male and female), and race (calculated variable for four race groups, categorized as White (non-Hispanic), Black (non-Hispanic), and Hispanic/other races). These categories allowed for a detailed examination of how demographic groups may be differently affected by the variables of interest. Socioeconomic characteristics were also a critical part of the analysis. Education (EDUCA) was categorized into two main groups: basic education (never attended school or only kindergarten, Grades 1-8 (elementary), Grades 9-11 (some high school), Grade 12 or GED (high school graduate)) and advanced education (1-3 years in college (some college or technical) and 4 years or more in college (college graduate)). Income (_INCOMG) is categorized as <$50,000 and >$50,000. Data from participants who responded to the selected disease, risk factor, and control variables were included in this study, ensuring completeness of data for analysis.

Descriptive statistics, including numbers and percentages, were generated for each variable using cross-tabulations in the BRFSS WEAT. The data were exported to Microsoft Excel (Microsoft Corp., Redmond, WA, US) for organization and further processing. Statistical analysis was conducted using R version 4.3.1 (2023; R Foundation for Statistical Computing, Vienna, Austria, https://www.R-project.org/). Results were expressed as odds ratios (ORs) with 95% confidence intervals (CIs). A p-value less than 0.05 was considered statistically significant.

## Results

The 2022 data from the BRFSS were surveyed. Table 1 shows the prevalence of self-reported stroke among current smokers and non-smokers. Among 49,682 participants who reported smoking, 3,335 (6.7%) had a self-reported stroke. In contrast, among 358,724 participants who did not smoke, 344,284 (96%) did not report having had a stroke. Overall, participants who smoked were 1.716% more likely to report having had a stroke compared to non-smokers.

**Table 1:** Prevalence of current smokers among participants with self-reported stroke *Significant.

		Stroke		OR (95% CI)
		Yes	No	
Current smoker	Yes	3,335 (6.7%)	46,347 (93.3%)	1.716* (1.6498, 1.7839)
	No	14,440 (4%)	34,4284 (96%)	

Table 2 shows the prevalence of self-reported stroke among participants who smoke: 1.6% in the 18-24 age group, 2.6% in the 25-44, 7.4% in the 45-64, and 11.1% in those aged 65 and above. By gender, self-reported stroke was slightly less common in male smokers (6.5%) than in female smokers (6.9%). In terms of race, 6.5% of White smokers reported stroke, compared to 9.6% of Black smokers and 6.1% of Hispanic smokers.

**Table 2:** Prevalence of current smoking among participants with self-reported stroke, stratified by demographic characteristics *Significant.

Demographic characteristics	Current smoker	N	Participants with stroke	Participants without stroke	OR (95% CI)
Age groups					
18-24 years	Yes	1,604	26 (1.6%)	1,578 (98.4%)	5.233* (3.1999, 8.3171)
	No	23,263	73 (0.3%)	23,190 (99.7%)	
25-44 years	Yes	14,589	385 (2.6%)	14,204 (97.4%)	3.231* (2.8411, 3.669)
	No	83,532	695 (0.8%)	82,837 (99.2%)	
45-64 years	Yes	21,321	1,570 (7.4%)	19,751 (92.6%)	2.373* (2.2313,2.5227)
	No	114,955	3,726 (3.2%)	111,229 (96.8%)	
65+ years	Yes	12,168	1,354 (11.1%)	10,814 (88.9%)	1.599* (1.5048,1.6984)
	No	13,6974	9,946 (7.3%)	127028 (92.7%)	
Gender					
Male	Yes	25,176	1,632 (6.5%)	23,544 (93.5%)	1.639* (1.5495,1.7335)
	No	167,766	6,806 (4.1%)	160,960 (95.9%)	
Female	Yes	24,506	1,703 (6.9%)	22,803 (93.1%)	1.793* (1.6977, 1.8938)
	No	190,958	7,634 (4%)	183,324 (96%)	
Race					
White, non-Hispanic	Yes	35,117	2,276 (6.5%)	32,841 (93.5%)	1.614* (1.5397, 1.6912)
	No	262,155	10794 (4.1%)	251,361 (95.9%)	
Black, non-Hispanic	Yes	4,404	424 (9.6%)	3,980 (90.4%)	1.915* (1.7049, 2.1465)
	No	26,958	1,421 (5.3%)	25,537 (94.7%)	
Hispanic/others	Yes	8,615	525 (6.1%)	8,090 (93.9%)	2.158* (1.948, 2.3872)
	No	59,528	1,738 (2.9%)	57,790 (97.1%)	

Table 3 shows that the prevalence of self-reported stroke among participants who smoke is 7.5%, while 5.3% of participants with advanced education reported a stroke. When stratified by income, self-reported stroke is more common among smokers with an annual income of <$50,000 (8.7%), compared to 3.2% among those earning >$50,000. The likelihood of association between smoking and self-reported stroke is 1.456% in participants with basic education and 1.728% in those with advanced education. Similarly, the likelihood of association is 1.457% among smokers earning <$50,000 and 1.299% in those with incomes >$50,000.

**Table 3:** Prevalence of current smoking among participants with self-reported stroke by socioeconomic characteristics *Significant.

Socioeconomic characteristics	Current smoker	N	Participants with stroke	Participants without stroke	OR (95% CI)
Educational level					
Basic education	Yes	23,739	1,781 (7.5%)	21,958 (92.5%)	1.456* (1.3761,1.5399)
	No	98,239	5,184 (5.3%)	93,055 (94.7%)	
Advanced education	Yes	25,787	1,541 (6%)	24,246 (94%)	1.728* (1.6332,1.8265)
	No	259,073	9,193 (3.5%)	249,880 (96.5%)	
Annual income					
	Yes	25,783	2,251 (8.7%)	23,532 (91.3%)	1.457* (1.3855, 1.531)
	No	112,050	6,905 (6.2%)	105,145 (93.8%)	
>$50,000	Yes	16,355	529 (3.2%)	15,826 (96.8%)	1.299* (1.183, 1.4238)
	No	179,802	4,511 (2.5%)	175,291 (97.5%)	

## Discussion

This retrospective study analyzed 2022 BRFSS data to assess the association between smoking and stroke. Findings indicate that current smokers have a significantly higher risk of stroke compared to non-smokers.

Smokers exhibited 71% higher odds of developing stroke than non-smokers. Socioeconomic factors influenced this relationship, with a greater risk observed in lower-income groups compared to higher-income individuals.

These findings suggest a complex relationship between smoking and stroke, influenced by demographic and socioeconomic factors. Overall, smoking is associated with an increased risk of stroke.

The overall prevalence of stroke among smokers was 6.7%, compared to 4% in non-smokers. This aligns with previous studies, including one showing that even smoking one cigarette daily increases stroke and heart disease risk [16]. Another study demonstrated that smoking cessation lowers cardiovascular risk within five years, though former smokers still face elevated risk compared to never-smokers [17].

Age, gender, and ethnicity further modulated stroke risk. As shown in Table 2, smokers aged 18-24 years had a markedly higher stroke risk than their non-smoking peers, with a nearly 5-fold increase. Female smokers had a 79% higher risk compared to female non-smokers, and Hispanic/other ethnic group smokers exhibited the greatest increase in stroke odds (115% higher) relative to non-smokers. Risk varied by age group: 59% higher in those aged 65 and older, 13.7% higher in the 45-64 age group, and 22.3%-42.3% higher in ages 18-24.

These findings are consistent with prior research suggesting smoking significantly elevates stroke risk until around age 50 [18].

Socioeconomic disparities were evident. Table 3 shows that smokers with basic education had 45.6% higher odds of stroke; those with advanced education had 72.8% higher odds; and smokers earning <$50,000 annually had 45.7% higher stroke risk, while those earning >$50,000 had 29.9% higher odds. These trends align with studies showing stroke disproportionately affects socioeconomically disadvantaged populations, even within high-income countries [19]. Moreover, low educational attainment is associated with higher mortality, recurrent stroke, and cardiovascular complications following ischemic stroke [20].

In summary, this study highlights a clear association between smoking and increased stroke risk, influenced by age, sex, race/ethnicity, income, and education. These findings suggest that the link between smoking and stroke is shaped by a complex interplay of behavioral, socioeconomic, and possibly biological factors. Well-designed prospective studies are needed to further clarify the mechanisms involved and help develop targeted prevention strategies.

Limitations

This study has several limitations. As a retrospective observational analysis, it identifies associations but cannot establish causation. Self-reported data introduce potential recall and social desirability bias. Data collection via telephone surveys may lead to selection bias. The BRFSS dataset lacks clinical details such as stroke type, severity, and timing, which limits interpretation of outcomes and risk patterns. These limitations highlight the need for longitudinal studies with objective measures and detailed clinical data to better understand how smoking and other factors affect stroke risk and recovery.

## Conclusions

A retrospective study was conducted using the 2022 BRFSS database to assess the association between smoking and stroke in the US. The findings revealed that smokers across various demographic and socioeconomic groups had an increased likelihood of experiencing stroke. However, this risk appeared relatively lower among older adults, males, White individuals, and those with higher income or basic education levels. To better understand these patterns, future research should prioritize prospective studies aimed at exploring the underlying mechanisms behind these associations. Such studies could help determine whether smoking itself, or other confounding factors, contributes more significantly to the elevated stroke risk. Insights from this research may inform the development of targeted prevention and intervention strategies. Considering lifestyle and sociodemographic factors will be crucial for designing effective public health approaches. While this study focused on stroke, the findings may also have broader implications for other health conditions, such as melanoma, by emphasizing the need to account for population diversity in disease prevention efforts.
